# Glycosylation Profile of the Transferrin Receptor in Gestational Iron Deficiency and Early-Onset Severe Preeclampsia

**DOI:** 10.1155/2019/9514546

**Published:** 2019-02-03

**Authors:** Alejandra María Gómez-Gutiérrez, Beatriz Elena Parra-Sosa, Julio Cesar Bueno-Sánchez

**Affiliations:** ^1^Professor Facultad de Medicina, Departamento de Fisiología y Bioquímica, Grupo Reproducción, Universidad de Antioquia, Carrera 53 # 61-30, Torre 2, Laboratorio 534, Medellín, Colombia; ^2^Grupo de Investigación Alimentación y Nutrición Humana (GIANH-END-UdeA), Universidad de Antioquia, Calle 62 No 52-59, Torre 1, Laboratorio 413, Medellín, Colombia

## Abstract

**Objective:**

To examine the expression of hypoxia-inducible factor-1*α* (HIF-1*α*), TfR1, and TfR1-attached terminal monosaccharides in placentas of women with IDAP and severe preeclampsia.

**Methods:**

TfR1 and HIF-1*α* were detected by western blot. Immunoadsorption of TfR1 was performed to characterize the terminal monosaccharides by specific lectin binding.

**Results:**

There was no difference in the expression of TfR1 and HIF-1*α* between groups. Lectin blot analysis pointed out an overexpression of galactose *β*1-4* N*-acetylglucosamine (Gal-GlcNAc) and mannose in severe preeclampsia.

**Conclusion:**

The increase in Gal-GlcNAc may be due to the increased presence of antennary structures and the mannose glycans of TfR1 may indicate the presence of misfolded or incomplete proteins. These findings may be associated with the low expression of placental TfR1 in women with preeclampsia.

## 1. Introduction

Iron is an essential nutrient for metabolic processes [1]. Iron deficiency anemia in pregnancy (IDAP) is diagnosed by a low hemoglobin concentration (<11 g/dL) and a reduction of maternal serum ferritin (<15 *μ*g/L). This condition affects 48% of women in the world [2], with deleterious consequences in the maternal and fetal health. IDAP decreases the work capacity, increases the risk of infection, and requires a longer recovery time during pregnancy [3, 4]. Additionally, IDAP has been associated with intrauterine growth restriction (IUGR), low birth weight (LBW), and preterm delivery [5, 6]

On the other hand, disturbances in iron status related to hypertensive disorders in pregnancy, particularly preeclampsia, have been reported [7–9]. Preeclampsia is a proinflammatory condition characterized by hypertension and a target organ injury, associated with a shallow trophoblast invasion of the spiral arteries [10]. In this condition a disturbance in the maternal values of plasmatic ferritin (> 41 *μ*g/L), impaired placental bed perfusion and similarly, preterm delivery, IUGR, and LBW has been reported [11].

In pregnancy, a large amount of iron is transferred from the mother to the fetus across the placenta [12, 13], which is captured by the transferrin receptor (TfR1) in the apical membrane of the syncytiotrophoblast (STB) [14]. In previous studies, an increase of placental TfR1 expression in IDAP was observed [15–18] as a possible compensatory mechanism in iron uptake to protect the fetus. On the contrary, a reduced expression of TfR1 was noted in preeclampsia, which could contribute to IUGR in most of these pregnancies [19, 20].

The prolonged status of placental hypoxia in both, preeclampsia and IDAP, increases the accumulation of the *α* subunit of the transcriptional factor induced by hypoxia (HIF-1*α*) [21]. The HIF-1*αβ* heterodimer is stabilized by hypoxia and promotes the binding to hypoxia response elements (HRE) in target genes [22]. Transcriptional products of these genes are responsible for changes in the cellular phenotype, and therefore since the promoter of TfR1 has an HRE, it is susceptible to transcriptional control by HIF [23]. This finding would suggest that in women with preeclampsia, who have placentas under the prolonged effect of hypoxia [24], this could increase the levels of TfR1; however, the evidence regarding a reduction of TfR1 in preeclamptic placentas raises the possibility that this mechanism of gene-transcriptional control fails in this disease.

TfR1 is a glycoprotein composed of two 85 kDa monomers linked by two disulfide bonds. The sequence of each subunit has 760 amino acid residues: 61 residues in the N-terminal cytoplasmic domain, 28 residues in the hydrophobic transmembrane domain, and 672 residues in the extracellular domain [25]. In the extracellular domain, TfR1 contains three* N*-linked glycan chains [26], and one* O*-linked oligosaccharide [27]. It is possible that alteration in the expression of TfR1 is due to posttranslational modifications associated with the glycosylation process. Structural and functional analyses of glycans from TfR1 have shown that glycosylation is not required for dimerization and binding with transferrin but that the affinity may be influenced by the presence or absence of a specific glycan [28]. Failure of TfR1 glycosylation reduces the export of TfR1 to the cell membrane [28], because the* N*-glycans appear to be involved in proper folding of the protein [29]. Furthermore, the removal of* O*-glycosylation increases the susceptibility of TfR1 to proteolysis, generating a soluble form of TfR1 [30].

Previously, we found changes in the glycosylation profile of trophoblastic villi in women with preeclampsia compared with normal gestation [31]. In the present study, we sought to investigate whether changes occur in the glycosylation profile of TfR1 in placentas from women with preeclampsia.

## 2. Materials and Methods

### 2.1. Subjects

The sample size was estimated for convenience (6 placentas in each group). The placentas of each group (control, IDAP, and early-onset severe preeclampsia) were obtained from women undergoing elective caesarean, aged 18 to 40 years, with monofetal pregnancy in the third trimester and a normal or overweight gestational body mass index (BMI) [32]. Women's weight and height were taken with precision equipment. The measurements of weight and height of women with preeclampsia were obtained from clinical data. Women in the control group had adequate iron status and did not have preeclampsia.

The following criteria of the American Congress of Obstetricians and Gynecologists were considered to diagnose a woman with early-onset severe preeclampsia (≤ 34 weeks): (1) systolic blood pressure of 160 mmHg or higher, or 110 mmHg diastolic blood pressure or higher in two occasions at least 4 hours apart while the patient was lying on a bed resting; (2) proteinuria of 5 gr or higher in a 24-h urine collection; (3) oliguria of less than 500 mL of urine in 24 hours; (4) thrombocytopenia (platelets <100,000/ml); (5) no patients were in labor or presented a HELLP syndrome (Hemolysis, elevated liver enzymes, low platelet count) at the time of sample collection [33]. Women with preeclampsia had no altered state of iron. Severely preeclamptic women returned to normal blood pressure values twelve weeks postpartum.

IDAP pregnant women did not have preeclampsia. The iron deficiency anemia was diagnosed according to the WHO criteria [2]: hemoglobin <11 g/dL and serum ferritin <15 *μ*g/L.

The exclusion criteria were patients diagnosed as underweight or obese, preexisting proteinuria and hypertension, previous pregnancies with genetic or congenital malformation, renal disease, infections or proinflammatory states that appeared in the current gestation, diabetes mellitus, collagen disorders, autoimmune disease, cancer, or the use of psychoactive substances.

### 2.2. Determination of Hemoglobin, Ferritin and C-Reactive Protein

Serum ferritin was tested by the electrochemiluminescence immunoassay (ECLIA) in an automated analyzer (Roche Modular Analytics E170). The hemoglobin was tested by the cyanmethemoglobin method modified with automatic reading equipment (Abbott Cell Dyn 3700) in control and IDAP women groups. In the case of early-onset severely preeclamptic women, the hemoglobin value was obtained from medical records. For all groups C-reactive protein was measured by immunoturbidimetry, in an automated analyzer (Roche Modular P800), and used to properly interpret the results of maternal ferritin. When the C-reactive protein was higher than 1.5mg/dL thus the cut-off of serum ferritin concentration was below 30 *μ*g/L to classify an iron deficiency [34].

### 2.3. Processing of Placental Villi and Basal Plate Decidua

Placental samples were processed in the first hour after cesarean, according to the protocol of Kliman et al. modified by Maldonado [35]. The placental cotyledons were mechanically disaggregated after removing the decidua and trophoblastic villi were obtained and fetal vessels were discarded. Fragments of placental villi were stored at −70°C until processing. Trophoblastic tissue was washed with phosphate buffered saline (PBS), supplemented with 1% penicillin/streptomycin, and 250 *μ*g/mL of amphotericin (GIBCO-Invitrogen). From each sample, 200 mg of trophoblastic villi was mechanically homogenized and sonicated in buffer with 150 mM NaCl plus protease inhibitor (Protease Inhibitor Cocktail Calbiochem, 100 mM, bovine Aprotinin 80 *μ*M, Bestanin 5 mM, E-64 inhibitor 1.5 mM, Leupentin 2 mM, Tris HCl 1 M). Centrifugation was performed at 15000 rpm, 4°C, for 15 min and the protein extract was collected. The protein concentration was determined using the commercial kit™ Pierce BCA Protein Assay.

### 2.4. Western Blot

Electrophoresis was performed in 10% polyacrylamide gels and 1% sodium dodecyl sulfate (SDS-PAGE). In loading buffer (0.5 M Tris-HCl, pH 6.8, 30% glycerol, 6% mercaptoethanol, 10% SDS, 0.01% bromophenol blue in distilled water), 40 *μ*g of protein was dissolved; protein separation was performed at 130V. The proteins were then transferred at 116 mA for 60 min onto PVDF membranes (BIO-RAD) using a semidry blotter with Towin buffer (methanol 20% v/v). The membranes were incubated overnight at 4°C with HIF-1*α* antibodies (1:500; BD Biosciences, Palo Alto, CA), TfR1 antibodies (1:500; CD71 (b3/25), Santa Cruz Biotechnology, Inc.), and anti-*β*-actin or anti-*α*-tubulin (1:1000; Calbiochem). The membranes were incubated for 60 min at room temperature with a secondary antibody conjugated to horseradish peroxidase; bands were visualized using enhanced chemiluminescence (SuperSignal West Pico, Thermo Scientific) and an X-ray film. The relative amount of target protein was expressed as a ratio to *β*-actin or *α*-tubulin analyzed by western blotting

### 2.5. Immunoadsorption of TfR1

In a 1.5 ml microcentrifuge tube 500-1000 *μ*g of protein extract was loaded. Primary antibodies (CD71 (b3/25), Santa Cruz Biotechnology, Inc.) were added (5-10 *μ*l/0.2-2 *μ*g) and the sample was incubated for 3 hours at 4°C. Protein A/G Plus-agarose (Santa Cruz Biotechnology, Inc.) was added (20 *μ*l) and incubated in an orbital shaker overnight. The immunoprecipitation was performed by centrifugation at 2,500 rpm for 5 min at 4°C and the resulting supernatant was discarded. The pellet was washed 4 times with 1.0 ml PBS by centrifugation and solubilized in 40 *μ*l of 1X electrophoresis sample buffer and the immunoprecipitated protein was loaded onto a gel for electrophoresis and western blotting. A mouse IgG1 monoclonal antibody against human actin was used as a control of immunoadsorption (Pierce Biotechnology).

### 2.6. Detection of Terminal Monosaccharides in TfR1

Lectin blot analysis was performed using the kit DIG Glycan Differentiation (Roche). After transfer, the membranes were blocked for 30 min (in 9 ml blocking solution 1X), and incubated for 1 hour with lectins coupled to digoxigenin. The lectins* Galanthus nivalis agglutinin* (GNA),* Sambucus nigra agglutinin* (SNA), and* Datura stramonium agglutinin* (DSA), which detect, respectively, mannose, *α*2-6-linked sialic acid, and Galactose *β*1-4* N*-acetylglucosamine (Gal-GlcNAc), were diluted to 0.1% v/v in buffer 1 (TBS; 1 mM MgCl_2_, 1 mM MnCl_2_, 1 mM CaCl_2_, pH 7.5). The lectins* Maackia amurensis agglutinin* (MAA) and* peanut agglutinin* (PNA), which detect, respectively, *α*2-3-linked sialic and Gal 1–3 Gal-NAc, were diluted to 0.5 and 1.0% v/v, respectively. Incubation with polyclonal sheep anti-digoxigenin antibodies conjugated to alkaline phosphatase 0.1% v/v was for 1 hour. The membranes were immersed in staining solution containing nitro blue tetrazolium/BCIP (5-bromo-chloro-3-indolyl phosphate), 2.0% v/v, prepared in buffer 2 (0.1 M Tris-HCl, 0.05 M MgCl_2_, 0.1 M NaCl, pH 9.5) for the detection of the lectin-glycan complex. When a dark precipitate was observed in the membrane, the reaction was stopped with distilled water. As load control, we ran samples in two gels in the same electrophoretic chamber: one of these was used for lectin blot assays and the other one was transferred to PVDF membrane in order to detect the immunoabsorbed TfR1 (40ug) (Supplementary figure 1).

### 2.7. Statistical Analysis

The intensity and the area of the protein and glycan bands bound to them were calculated by Image J software, version 1.44. For the WB, a high resolution scanned images of X-ray film obtained with HP Scan and Capture® software, version 40.0.245.0 (16-bit tiff images), were used for densitometry and normalization. For the LB a densitometry of the glycan bands and a normalization were performed with the IgG antibody used for immunoadsorption. The groups were compared with the Kruskal-Wallis test for data with a nonparametric distribution. The data are presented as medians and their ranges. The analyzes were done using the GraphPad PRISM 5.0® program (Software Inc., La Jolla, Ca, USA).

## 3. Results

### 3.1. Sociodemographic Characteristics of the Samples

Table 1 summarizes the clinical characteristics of the 3 groups of women, which correspond to the inclusion criteria. Women with IDAP showed both lower ferritin (*P *= 0.0026) and hemoglobin values (*P* = 0.0060) and the group of women with preeclampsia showed the highest values in systolic and diastolic blood pressure (*P* = 0.0047 and* P* = 0.0036). The gestational age was lower in the group of women with preeclampsia (*P* = 0.0023), due to the severity of the signs that prematurely forced to terminate pregnancy in all cases. The maternal age and the number of pregnant and maternal BMI showed no differences between the groups.

### 3.2. Protein Expression

**Figure 1:**
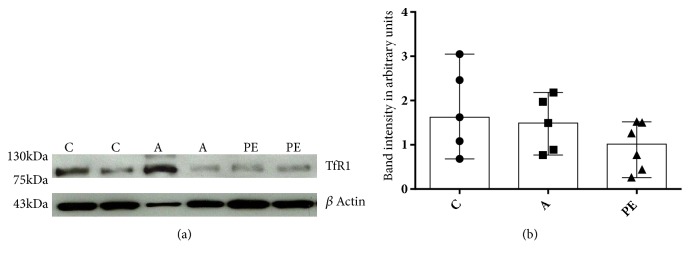
TfR1 expression in placental villi. (a) Representative image of a Western blot (an entire picture is presented in the supplementary figure 4A). (b) Expression of TfR1 in trophoblastic villi in the control group (C) n=5, the group with IDAP (A) n=5 and the group with early-onset severe preeclampsia (PE) n=6. No statistically significant difference in protein expression in the three groups of women was detected. The data are shown expressed as median and range, Kruskal-Wallis statistical analysis.

Iron uptake by the placenta is performed by TfR1 anchored in the apical membrane of the SBT. The expression of TfR1 was tested in microvilli obtained from pregnant women; no significant differences between groups were noted (Figures 1(a) and 1(b)).

**Figure 2:**
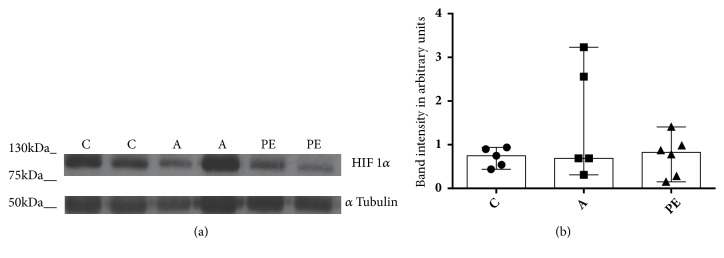
HIF-1*α* expression in placental villi. (a) Representative image of a Western blot (an entire picture is presented in the supplementary figure 4B). (b) Expression of HIF-1*α* in trophoblastic villi in the control group (C) n=5, the group with IDAP (A) n= 5 and the group with early-onset severe preeclampsia (PE) n=6. No statistically significant difference in protein expression between the three groups of women was observed. The data are shown as median and range, Kruskal-Wallis statistical analysis.

Preeclamptic placenta has been associated with an exacerbated hypoxic state; however, there was no statistically significant difference in the expression of HIF-1*α* in the groups examined here (Figures 2(a) and 2(b)).

### 3.3. Immunoadsorption of TfR1

To determine the glycosylation profile of TfR1 of the three groups of women, the protein was immnunoabsorbed. Two bands were observed (Supplementary Figure 2), possibly due to the presence of differentially glycosylated forms of TfR1: a lower molecular weight protein (approximately 85 kDa) and a higher molecular weight protein (approximately 91 kDa), as it was described by Georgieff in placental tissues [36].

### 3.4. Characteristics of the Placental TfR1

**Figure 3:**
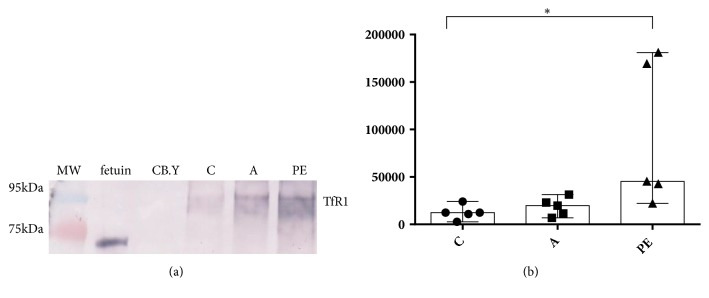
Expression pattern Gal-GlcNAc detected by the DSA lectin. (a) Representative image of a lectin blot; as positive control fetuin was used and as negative control carboxypeptidase Y (CB.Y). (b) Expression of the Gal-GlcNAc pattern of TfR1 in trophoblastic villi in the control group (C), the group with IDAP (A), and the group with early-onset severe preeclampsia (PE). The data are presented as median and range, Kruskal-Wallis statistical analysis, where p <0.05 *∗*.

Differential expression of terminal glycans was determined by the relative intensity of lectin blot patterns calculated by densitometry. Gal-GlcNAc, detected by DSA lectin, showed an increased relative expression level (*P* <0.05) in placental TfR1 of women with preeclampsia compared to the IDAP and healthy control groups (Figures 3(a) and 3(b)).

**Figure 4:**
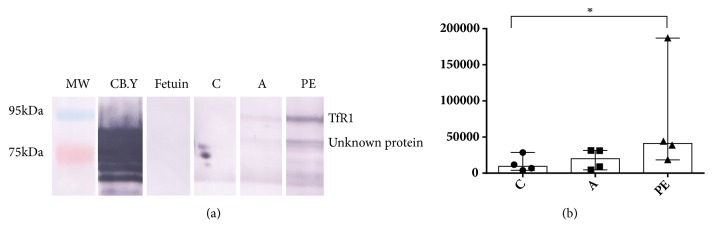
Expression pattern of mannose detected by the GNA lectin. (a) Representative image of a lectin blot; as positive control carboxypeptidase Y (CB.Y) was used, and as negative control fetuin. (b) Expression of the mannose pattern of TfR1 in trophoblastic villi in the control group (C), the group with IDAP (A), and the group with early-onset severe preeclampsia (PE). The data are presented as median and range, Kruskal-Wallis statistical analysis, where p <0.05 *∗*.

The pattern of terminal mannose was significantly higher (*P* <0.05) in the group of women with preeclampsia compared with the IDAP and control groups. Additionally, terminal mannose was observed in a band of approximately 70 kDa only in the group of women with preeclampsia, but was not detected in any of the other groups (Figures 4(a) and 4(b)). Moreover, this band did not appear in the immunoblot of TfR1.

No statistical difference in the expression of *α*2-3-linked sialic acid and Gal 1–3 Gal-NAc was observed comparing the groups, although the group of women with preeclampsia had a tendency to increase the expression of sialic acid *α*2-3 (Supplementary Figure 3).

The expression of *α*2-6-linked sialic acid was not detected by SNA lectin in the assays performed, indicating the absence of the monosaccharide in TfR1 (data not shown).

## 4. Discussion

The placenta is a temporary organ emerging as promoter of the interchange of nutrients and waste products between maternal and fetal circulation. The STB transfers nutrients to the fetus by paracellular and transport mechanisms such as simple and facilitated diffusion, carrier-mediated transport, or those transported by receptor-mediated membrane vesicles, as is the case of TfR1 [37, 38].

As previously mentioned, IDAP and preeclampsia present disturbances in the expression of TfR1. In our study, we did not find significant changes in the expression of TfR1 between groups of women. In the case of women with IDAP, this can be attributed to a brief period of the adverse effects of iron deficiency. In this sense Zhang et al. [39] found a higher occurrence of preterm delivery in women with IDAP in the first weeks of gestation, while those with anemia that occurred in the last trimester of pregnancy showed a reduction in the risk of complications, particular in preterm delivery. Thus, the decisive events to produce changes in the expression of placental proteins such as TfR1 could be modulated by the time of the iron deficiency; in other words, fetal iron uptake must be reduced only in a chronic state of anemia, when the placental storage of iron is lacking [40].

Previous studies suggested that enhanced TfR1 expression in a preeclamptic placenta would be highly likely because the TfR1 gene possesses an HRE [41, 42]. In preeclamptic placenta, prolonged hypoxia beyond the first trimester has been demonstrated [22, 43] and HIF-1*α* is abnormally elevated [44, 45]. However, we did not find statistically significant changes in the expression of TfR1 in preeclamptic women, nor did we find statistically significant changes in the expression of HIF-1*α*. Because TfR1 is a product of the signaling pathway of HIF-1*α* [23], it is possible that the similarity in expression pattern of HIF-1*α* explains the same expression of TfR1 in the 3 groups of women studied. This can be explained since the degree of hypoxia in women with preeclampsia and IDAP is not higher than in the control group. Huppertz et al. [46], have argued that the origin of preeclampsia is not due to a failure of trophoblast invasion causing hypoxia, but instead to a disturbance in the development of trophoblasts that affects differentiation. Thus, our results can be interpreted in the same sense as proposed by Huppertz et al, and it can be considered that women with IDAP or preeclampsia do not present a HIF-1*α*-activated signaling pathway in the third trimester of gestation.

Khatun et al. [19], observed downregulation of placental TfR1 in preeclamptic women; in others studies this downregulation was attributed to the intracellular iron concentration [47]. Thus, we found changes in the nutrient status, as demonstrated by the high concentrations of serum ferritin 92.5 ng/ml (Table 1), indicating a disturbance in the nutrient homeostasis in this group of women. It is known that serum ferritin is an acute phase reactant and therefore the increase in ferritin observed in the preeclamptic women can be associated with the inflammation observed in this disease [48]. Additionally, pregnancy has been defined as a systemic inflammatory condition, and an increase in markers such as C-reactive protein has been reported [49]. In our research, there was a significant difference in the concentration of C-reactive protein between the group of healthy pregnant women and the group of pregnant women with preeclampsia (0,2-0,42 mg/dL and 0,68-1,98 mg/dL, respectively), suggesting that the inflammatory condition may be greater in preeclampsia.

Glycoproteins occupy a central position in the metabolism and in cellular interactions; in the case of placental TfR1, the role of post-translational modifications or differences in the glycosylation patterns in pregnancy has been poorly explored. Orberger and associates [50], tested the TfR1 glycan structures and found complex* N*-glycans with bi- and triantennary forms, some of them with antennas rich in lactosamine. The antennas presented mostly *α*2-3-linked sialic acid ends.

In this study, we undertook to characterize the glycosylation patterns of TfR1 expressed in the placenta of women with pregnancy disturbances and healthy women using lectins. In the group of women with preeclampsia, some assays made it possible to determine TfR1's overexpression of the Gal-GlcNAc and mannose terminal patterns detected by the DSA and GNA lectins, respectively. In the group of women with IDAP, there was no change compared with the control group. We did not find reactivity against sialic acid using the SNA lectin.

It is noteworthy that in the group of women with IDAP we did not find differences in the expression of different glycosylation patterns when compared with the other groups. Although expression changes were not detected in this study, functional measurements such as uptake and transport of iron by trophoblast cells should be performed in order to characterize the role of TfR1 in the pathophysiology of IDAP.

With regard to the glycosylation profile, we consider that DSA lectin binding without pretreatment of glycoproteins with neuraminidase, indicates the presence of nonsialylated glycans elongated by antennas. These glycans can be complex, bi or triantennary, as described by Orberger et al. [50], or also tetra-antennary. It could also indicate the presence of hybrid glycans in the three groups of women [51]. The overexpression of this glycosylation pattern in women with preeclampsia could be associated with the increased presence of antennas, possibly as a result of enhanced enzyme activity. Léger et al. [52], described the overexpression of triantennary glycans in the serum transferrin of pregnant women, which is possibly associated with increased activity of* N*-acetylglucosaminyltransferase IV and correlated with increased iron transport. The presence of antennas rich in lactosamine could play a role in intracellular trafficking of the transferrin-iron-TRf1 complex. Assays conducted by Carlsson et al. [53], indicate that galectin-3 binds to serum transferrin and this union seems to be decisive for the binding and subsequent trafficking with the complex. One can also consider that the expression of polylactosamine-enriched antennas in TfR1 plays a role in recognition of the complex by galectin-3.

The union of the GNA lectin, indicates the presence of mannosylated oligosaccharides that could be hybrids [54] in the placental samples of these groups of women. Additionally, the overexpression pattern of terminal mannose in women with preeclampsia may include oligosaccharides with polymannoses (Glc_1_Man_9_GlcNAc_2_, Man_9_GlcNAc_2_ and the three distinct isomers of Man_8_GlcNAc_2_), which could be an indication of an incompletely folded protein [55]. In the glycosylation process, a precursor oligosaccharide (Glc_3_, Man_9_, GlcNAc_2_) is transferred to the nascent glycoproteins in the endoplasmic reticulum (ER) [56]. This precursor is subsequently trimmed or elongated by the action of different enzymes, to lead to one of three types of* N*-glycans (highly mannosylated, hybrid or complex). As described above, intermediate products of *α* mannosidase provide signals to retain proteins and prevent further processing by the secretory pathway, as it can happened in the stress of the ER [55]. These proteins are retained by chaperones such as binding protein (BiP) or GRP78, a member of the family of heat shock proteins located in the ER, that binds to incomplete or misfolded proteins [57]. In the group of women with preeclampsia, it is likely that placental abnormalities could lead to malfunction of the ER, resulting in misfolding of proteins [58], in this case TfR1. To prevent the progression of defective TfR1 along the secretory pathway, TfR1 must be retained by proteins such as BiP, that could have co-precipitated, and explain the unknown protein band observed when using the GNA lectin. In mutagenesis assays, Williams and Enns [57], reported a band of about 78 kDa when immunoprecipitating TfR1. They showed that this protein which co-precipitated with mutated TfR1 was BiP. We indicate this here, but we did not establish which protein co-precipitated with TfR1.

Placental TfR1 expressed *α*2-3 sialic acid instead of *α*2-6 sialic acid and a tendency of overexpression of *α*2-3 sialic acid was observed in preeclamptic placentas. According with our previous findings, placental protein extracts from preeclamptic placentas are highly sialylated [31], including those linked to *α*2-6 position. Van den Eijnden et al. have proposed a low expression of *α*2-6 sialyltransferase as a possible explanation of the reduced *α*2-6 sialic acid in the TfR1 glycoprotein [59]. Another alternative hypotheses could be a conformational change of the tertiary structure of TfR1 that reduces the interaction with the *α*2-6 sialyltransferase as happend with other proteins [60] or increases the interaction with the specific sialydase [61].

The reduction of *α*2-6 sialic acid can promote an increase of galectin 3 binding to terminal galactose in TfR1 and the formation of the Transferrin-iron-TfR1 complex in the iron uptake process [53, 62]. In addition, an overexpression of *α*2-3 sialic acid in TfR1 of preeclamptic placentas can be associated with the resistance to the cleavage from the cell membrane [63] in accordance with the assays reported by Rutledge in which *α*2-3 sialic acid removal released TfR1 protein towards the culture media.

The differences in the glycosylation pattern of TfR1 in the group of women with preeclampsia may have implications for a wide variety of characteristics of the protein that in turn can affect its functionality. The differential binding of terminal monosaccharide residues in the oligosaccharide chains in the group of women with preeclampsia can contribute to changes in the mass, loading, and tertiary structure of the protein by steric effects in the molecule [64]. These changes could modify export of TfR1 to the cell membrane or modify its ligand affinity, events that could affect iron uptake by the placenta that in turn could modify the fetal nutritional status.

## Figures and Tables

**Table 1:** Demographic and clinical characteristics of women.

**Variable**	**Control**	**IDAP**	**Preeclampsia**	**Statistical significance**
	**n=6**	**n=5**	**n=6**	
Age (years)	26.5 (20 - 39)	24 (20 - 24)	26.5 (18 - 35)	*p 0.0943*
Gravid	2 (1 - 4)	3 (1 - 3)	1.5 (0 -3)	*p 0.3382*
Gestational age (weeks)	39 (37 - 39) *∗∗*	38 (37 - 39)	31.5 (30 - 33)*∗∗*	*p 0.0023*
BMI (kg/m^2^)	28.2 (25 - 30)	26.3 (24.6 – 26.7)	27.9 (24 – 30.4)	*p 0.1802*
Maternal hemoglobin (g/dL)	13.4 (11.9 - 15.1)*∗∗*	10.6 (9.6 – 10.9)*∗∗* *∗*	13.2 (11 - 15)*∗*	*p 0.0060*
Maternal ferritin (ng/mL)	27.3 (20 – 36.5)	7.8 (7.4 – 12.2)*∗∗*	92.5 (21.9 – 343.1)*∗∗*	*p 0.0026*
Systolic blood pressure (mmHg)	113.5 (110 - 135)*∗*	116.5 (105 - 120)*∗* *∗*	166.5 (150 - 200)*∗* *∗*	*p 0.0047*
Diastolic blood pressure (mmHg)	75 (70 - 83)*∗*	72 (70 - 81)*∗∗*	107 (94 - 120)*∗* *∗∗*	*p 0.0036*

Data are shown as median and range, Kruskal-Wallis statistical analysis, where *∗* p <0.05 and *∗∗* p <0.01.

## Data Availability

The data presented as medians and ranges from densitometry analysis used to support the findings of this study have been deposited in the URL https://www.dropbox.com/sh/txc43l7but8waxs/AACQTt9F_ifswletZMqhCOWMa?dl=0.

## Supplementary Materials

Supplementary Materials Supplementary Figure 1. Western blot of immunoabsorbed TfR1. As load control, we used two gels in the same electrophoretic chamber. One of these was used for lectin blot assays and the other one was transferred to PVDF membrane in order to detect the immunoabsorbed TfR1 (40ug). A. Representative image of Western blot assays. B. In accordance with the densitometric analysis there is no significant difference in immunoadsorbed TfR1 loaded. Control group (C), IDAP (A), and early-onset severe preeclampsia (PE). Supplementary Figure 2. Immunoadsorption of TfR1. Western blot of immunoprecipitated TfR1 of placental villi in the control group (C), IDAP (A), and early-onset severe preeclampsia (PE). A mouse IgG1 monoclonal antibody against human actin was used as a control of immunoprecipitation. Supplementary Figure 3. Expression of *α*2-3 linked sialic acid detected by the MAA lectin. A. Representative image of a lectin blot; as positive control fetuin was used and as negative control Asialofetuin. B. Expression of *α*2-3 linked sialic acid of TFR1 in trophoblastic villi in the control group (C), the group with IDAP (A), and the group with early-onset severe preeclampsia (PE). No statistical difference was found. Supplementary Figure 4. Representatives images of Western Blot of TfR1 and HIF-1*α*. Full films are presented. A. TfR1 and B. HIF-1*α*.
