# Supplementary material for: Glycosylation Profile of the Transferrin Receptor in Gestational Iron Deficiency and Early-Onset Severe Preeclampsia
Source: J Pregnancy. 2019 Feb 3;2019:9514546. doi: 10.1155/2019/9514546 (PMC6378037; doi:10.1155/2019/9514546)
Supplement: Supplementary Materials — Supplementary Figure 1. Western blot of immunoabsorbed TfR1. As load control, we used two gels in the same electrophoretic chamber. One of these was used for lectin blot assays and the other one was transferred to PVDF membrane in order to detect the immunoabsorbed TfR1 (40ug). A. Representative image of Western blot assays. B. In accordance with the densitometric analysis there is no significant difference in immunoadsorbed TfR1 loaded. Control group (C), IDAP (A), and early-onset severe preeclampsia (PE). Supplementary Figure 2. Immunoadsorption of TfR1. Western blot of immunoprecipitated TfR1 of placental villi in the control group (C), IDAP (A), and early-onset severe preeclampsia (PE). A mouse IgG1 monoclonal antibody against human actin was used as a control of immunoprecipitation. Supplementary Figure 3. Expression of α2-3 linked sialic acid detected by the MAA lectin. A. Representative image of a lectin blot; as positive control fetuin was used and as negative control Asialofetuin. B. Expression of α2-3 linked sialic acid of TFR1 in trophoblastic villi in the control group (C), the group with IDAP (A), and the group with early-onset severe preeclampsia (PE). No statistical difference was found. Supplementary Figure 4. Representatives images of Western Blot of TfR1 and HIF-1α. Full films are presented. A. TfR1 and B. HIF-1α. [file 9514546.f1.zip › 9514546.f1/Supplementary figure 4_JP_2648865.docx]

A


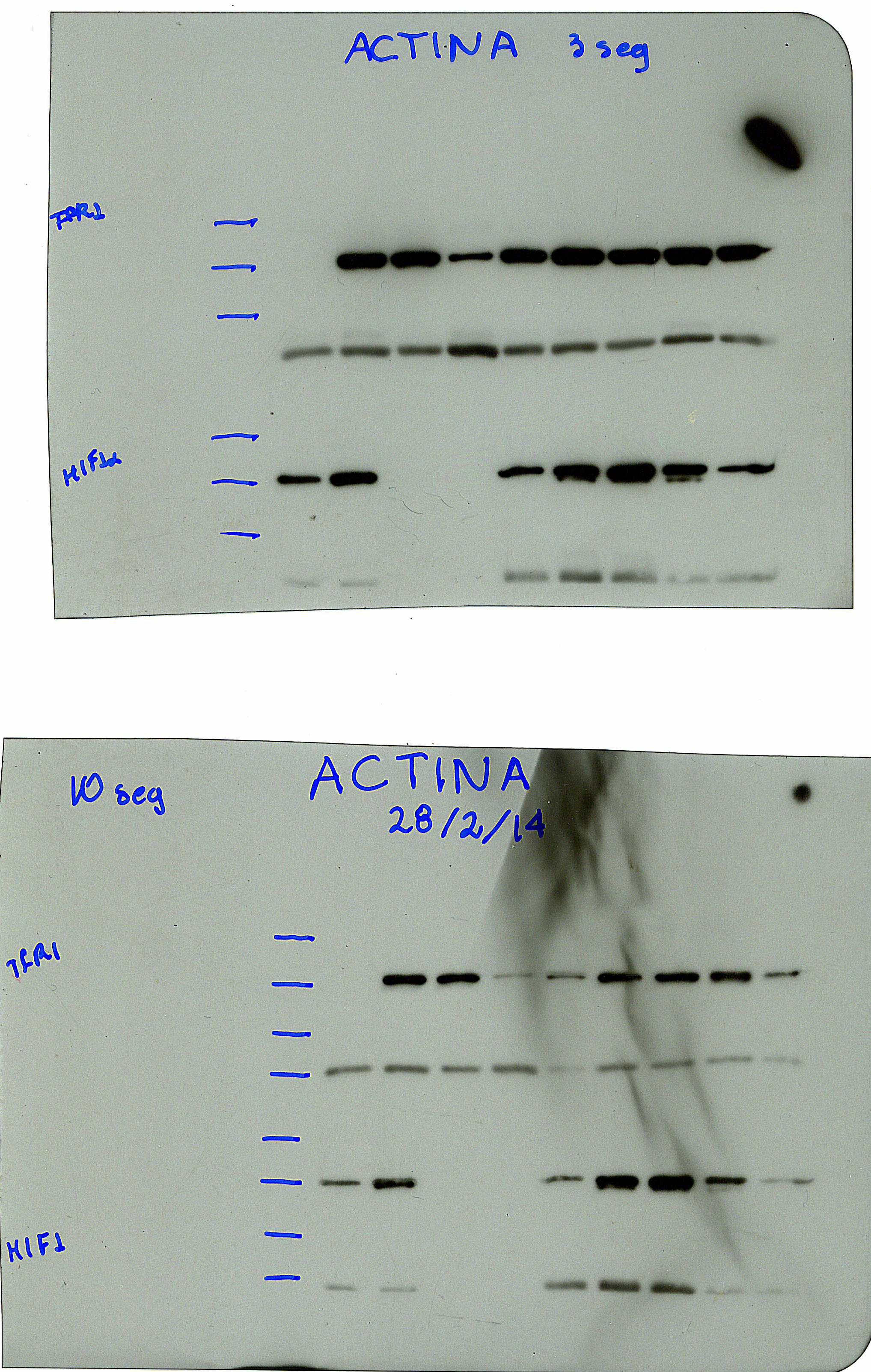


100 kDa_

75 kDa_

55 kDa_

MW Samples of TfR1 from placentas of pregnant women

TfR1

Β Actin

Brand

B


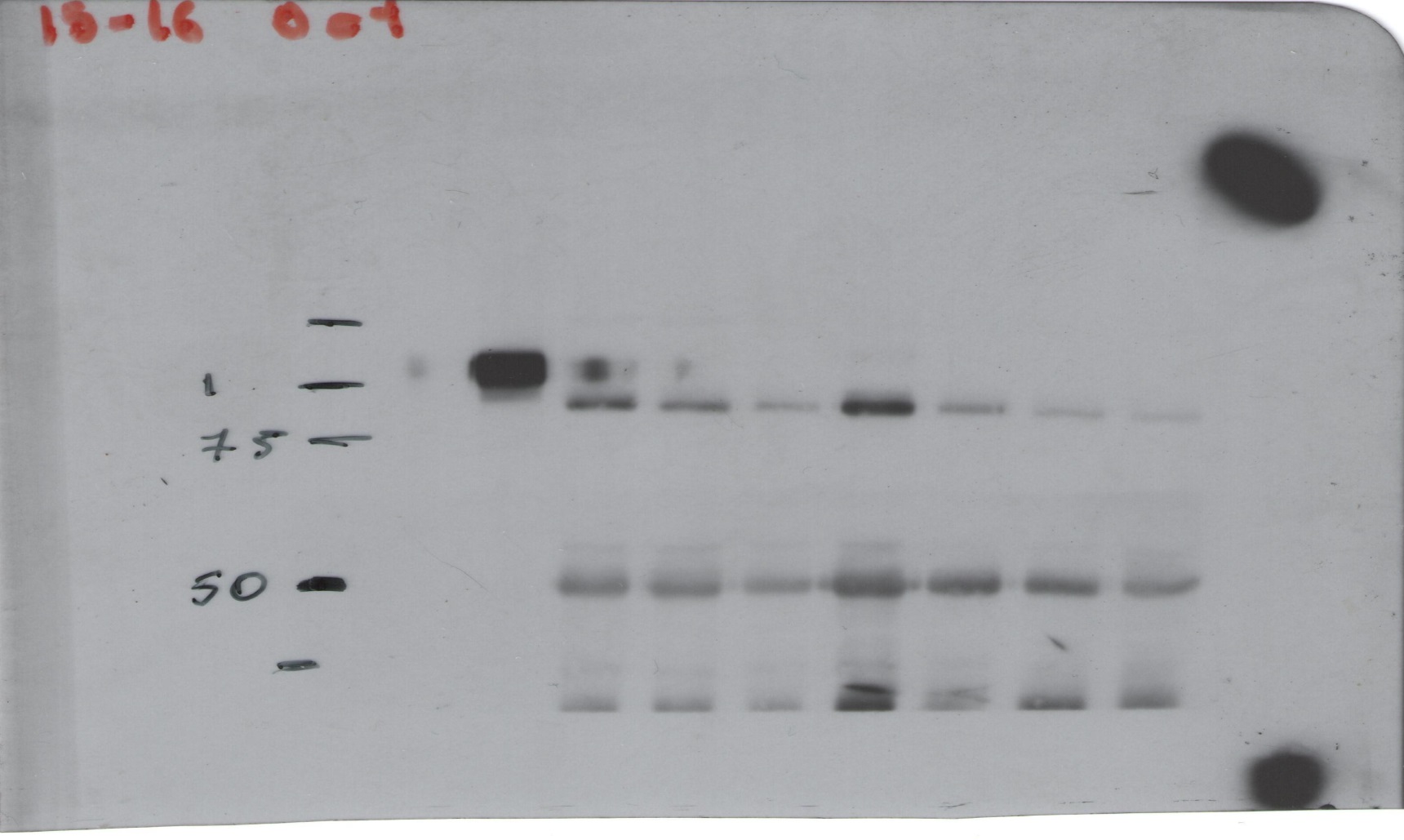


Brand

HIF1-α

α Tubulin

MW Samples of HIF1α from placentas of pregnant women

130 kDa_

75 kDa_

55 kDa_

Supplementary Figure 4. Representatives images of Western Blot of TfR1 and HIF1-α. Full films are presented. A. TfR1 and B. HIF1-α.
